# Prognostic Significance of the Systemic Inflammation Response Index (SIRI) in Patients with Hodgkin Lymphoma

**DOI:** 10.3390/medicina62020264

**Published:** 2026-01-27

**Authors:** Kadir Ilkkilic, Bayram Sen

**Affiliations:** 1 Department of Hematology, School of Medicine, Recep Tayyip Erdogan University, Rize 53200, Turkey; 2 Department of Biochemistry, Training and Research Hospital, Recep Tayyip Erdogan University, Rize 53200, Turkey; bayram.sen@saglik.gov.tr

**Keywords:** Hodgkin lymphoma, systemic inflammation response index (SIRI), prognosis

## Abstract

*Background and Objectives:* Interest in biomarkers reflecting the inflammatory nature of Hodgkin lymphoma (HL) is increasing. This study aimed to evaluate the prognostic significance of the Systemic Inflammation Response Index (SIRI) in patients with HL. *Materials and Methods:* In this study, 105 patients diagnosed with classical HL at the Hematology Clinic of Recep Tayyip Erdoğan University Faculty of Medicine between January 2015 and April 2025 were retrospectively evaluated. Patients were divided into 2 groups according to the SIRI cut-off value. *Results:* A high SIRI (≥3.78) was significantly associated with advanced disease stage, poor performance status, higher IPS-7 and IPS-3 scores, non-response or partial response to treatment, relapse, and increased mortality. A positive correlation was found between SIRI and IPS 7 scores (*p* < 0.001, rho = 0.355). In the univariate analysis for progression-free survival (PFS), hemoglobin, IPS 7 score, and SIRI were identified as prognostic factors; in the multivariate analysis, high SIRI was identified as an independent prognostic factor (*p* = 0.033). In the univariate analysis for overall survival (OS), age, hemoglobin, albumin, lymphocyte count, IPS 7 score, and SIRI were identified as prognostic factors; and, in the multivariate analysis, age over 45 and high SIRI were identified as independent prognostic factors (*p* = 0.016, *p* = 0.012). In the survival analysis, high SIRI levels were associated with shorter PFS and OS (*p* = 0.001, *p* < 0.001). Additionally, PFS and OS durations were shorter in patients with high IPS 7 scores (*p* < 0.001, *p* < 0.001). *Conclusions:* A high SIRI prior to treatment was identified as an independent prognostic factor in HL patients and was associated with shorter PFS and OS. This index may help identify high-risk patients and assist clinicians in their decision-making processes by enabling individualized risk assessment.

## 1. Introduction

Hodgkin Lymphoma (HL) is among the highly treatable malignancies, particularly in the young patient population, with a five-year relative survival rate reported to be approximately 90% [1]. However, despite generally good treatment response, clinical outcomes may still be poor in patients with primary refractory disease or those who relapse following salvage therapy, highlighting the importance of early risk stratification [2]. Despite the integration of novel agents into the early phases of treatment, the ability to identify patients at increased risk of relapse or disease-related mortality in advance is critically important for improving treatment responses, reducing treatment-related toxicity, and guiding the development of novel therapeutic strategies. Accordingly, the identification of prognostic factors remains a key clinical necessity [3,4,5].

The International Prognostic Score (IPS-7) and its simplified version, IPS-3, remain among the most widely used risk classification systems today for predicting survival in newly diagnosed advanced-stage HL patients [6,7]. However, with advances in treatment regimens and imaging techniques, findings have been reported indicating a decrease in the prognostic discriminatory power of the IPS-7 score [8,9]. Furthermore, even among patients classified in the same risk category, factors such as genetic heterogeneity, variability in response to treatment, and accompanying comorbidities can lead to significant differences in prognosis. In this context, various studies incorporating genetic characteristics, inflammatory biomarkers, and advanced imaging methods have been conducted to enable more detailed risk stratification and more accurate prediction of clinical course [10,11,12,13].

Systemic inflammation markers such as the neutrophil-to-lymphocyte ratio (NLR) and lymphocyte-to-monocyte ratio (LMR) have been reported to be associated with survival in HL. A recent study reported that high NLR and derived NLR were associated with shorter overall survival (OS) and progression-free survival (PFS) [14]. Another study showed that OS and event-free survival were shorter in patients with advanced HL who had an LMR below 2 before treatment [15]. In a study conducted on HL patients monitored with imaging-based treatment strategies, both NLR and LMR were shown to have significant prognostic value in predicting clinical outcomes [16]. Tumor-associated inflammatory processes have a decisive effect on the onset, progression, and response to treatment of the disease [17]. Studies conducted in various malignancies, including pancreatic cancer, gastric cancer, T-cell lymphoma, and HL, have highlighted the varying degrees of importance of systemic inflammatory markers in predicting disease prognosis [18,19,20,21].

The Systemic Inflammation Response Index (SIRI) is a new biomarker reflecting systemic inflammation based on monocyte, neutrophil, and lymphocyte counts. It has been studied in solid tumors, gastric lymphoma, peripheral T-cell lymphoma, and central nervous system lymphoma, and has been reported as a meaningful marker for predicting prognosis [22,23,24,25,26]. Macrophages, lymphocytes, cytokines, and neutrophils, which play an important role in the underlying tumor pathobiology and immune regulation in HL, contribute to an inflammatory microenvironment that supports tumor growth [27,28]. These tumor microenvironment-based predictive biomarkers may provide important information for prognosis prediction. In this context, SIRI may be considered a potentially useful index that could contribute to baseline risk assessment in patients with Hodgkin lymphoma; however, no comprehensive evaluation of its prognostic value in HL has been reported to date.

The aim of this study is to evaluate the prognostic significance of SIRI in HL patients.

## 2. Materials and Methods

### 2.1. Study Population

Between January 2015 and April 2025, 105 patients diagnosed with classical HL (cHL) at the Hematology Clinic of Recep Tayyip Erdoğan University Faculty of Medicine were retrospectively evaluated.

### 2.2. Inclusion Criteria

Patients aged ≥18 years with a histologically confirmed diagnosis of Hodgkin lymphoma, no history of other malignancies, and no prior exposure to chemotherapy or immunotherapy, who received curative-intent ABVD (doxorubicin 25 mg/m^2^, bleomycin 10 mg/m^2^, vinblastine 6 mg/m^2^, and dacarbazine 375 mg/m^2^ administered on days 1 and 15), with or without radiotherapy, were included in the study.

### 2.3. Exclusion Criteria

Patients with autoinflammatory diseases, active or chronic infections at the time of diagnosis, current steroid use, immunosuppressive therapy for any indication, or a first-line treatment regimen other than ABVD were excluded from the study.

All patients who met the predefined inclusion criteria were included in the final analysis, and no patients were excluded after the application of the inclusion and exclusion criteria.

Only patients with cHL were included in the study; nodular lymphocyte-predominant HL cases were excluded. Due to the limited number of patients within cHL histological subtypes, subgroup analyses based on histological classification were not performed.

### 2.4. Study Design and Data Collection

Patients clinical characteristics, follow-up information, and laboratory data were obtained retrospectively from hospital medical records. Age at diagnosis, gender, disease stage, Eastern Cooperative Oncology Group performance status (ECOG PS), presence of bulky disease (≥10 cm nodal mass), risk group, IPS-7 and IPS-3 scores, and comorbidities were recorded.

Laboratory data were obtained from blood samples collected after at least 12 h of fasting on the date of diagnosis, and white blood cell (Wbc), neutrophil, monocyte, lymphocyte, hemoglobin (Hb), platelet, albumin, direct bilirubin, and erythrocyte sedimentation rate (ESR) measurements were recorded.

Follow-up period, treatment response, relapse status, and mortality status were recorded.

Disease staging and treatment response were assessed according to the Lugano classification criteria using positron emission tomography—computed tomography (PET-CT) imaging [29]. Stage 1–2 cases were classified as early stage, while stage 3–4 cases were classified as advanced stage. Interim PET-CT was routinely performed in all patients after two cycles of ABVD chemotherapy.

Patients with advanced-stage disease who showed response after interim evaluation completed six cycles of ABVD, followed by end-of-treatment imaging for remission assessment.

Early-stage favorable risk patients received two cycles of ABVD followed by consolidative radiotherapy, after which treatment response was evaluated.

Early-stage unfavorable risk patients received two cycles of ABVD followed by interim evaluation; responders received an additional two cycles of ABVD (total four cycles) without radiotherapy, and response was reassessed at the end of treatment.

Risk grouping in early-stage patients was performed based on the German Hodgkin Study Group criteria. Accordingly, ESR > 50 mm/hour or ESR > 30 mm/hour in the presence of B symptoms, a mediastinal mass ratio >0.33, ≥3 involved lymph nodes, and the presence of extranodal involvement were evaluated. The presence of at least one of these factors was considered a unfavorable risk, while the absence of any of them was considered a favorable risk group [30].

IPS scores were calculated by assigning a point to each criterion. The IPS-7 score was calculated using the following criteria: male gender, stage IV disease, age ≥ 45 years, serum albumin level < 4 g/dL, Hb level < 10.5 g/dL, Wbc count > 15 × 10^3^/µL and/or absolute lymphocyte count < 0.6 × 10^3^/µL or constituting less than 8% of the total Wbc count. The IPS-3 score was determined using the following criteria: age ≥ 45 years, stage IV disease, and Hb level < 10.5 g/dL [6,7].

An IPS-7 score of 0–2 points was assessed as low risk, 3–4 points as moderate risk, and 5–7 points as high risk. An IPS-3 score of 0 points was assessed as low risk, 1–2 points as moderate risk, and 3 points as high risk.

SIRI was calculated using the formula: (Neutrophil count [10^3^/μL] × Monocyte count [10^3^/μL])/Lymphocyte count [10^3^/μL].

OS was defined as the time from diagnosis to death due to any cause or to the last follow-up date, while PFS was defined as the time from diagnosis to disease relapse or progression or to the earliest death.

Patients were divided into two groups based on the SIRI cutoff value determined by ROC analysis.

It should be noted that cutoff values for inflammation-based indices such as SIRI may be cohort-dependent and could vary across different populations. Therefore, external validation in independent patient cohorts is warranted to confirm the generalizability of these findings.

### 2.5. Statistical Analysis

All statistical analyses were conducted with IBM SPSS Statistics for Windows, Version 25.0 (IBM Corp., Armonk, NY, USA). The distribution of continuous parameters was analyzed using the Kolmogorov–Smirnov and/or Shapiro–Wilk tests. Continuous parameters with a normal distribution were presented as mean ± standard deviation, while those without a normal distribution were expressed as median (minimum-maximum). Categorical parameters were summarized as frequencies and percentages (%).

Comparisons of categorical parameters were carried out using the appropriate chi-square test or Fisher’s exact test. For comparisons between two independent groups, Student’s *t*-test was utilized for continuously measured parameters that followed a normal distribution, and the Mann–Whitney U test was applied for parameters that did not follow a normal distribution. Receiver operating characteristic (ROC) curve analysis was performed to determine the prognostic value of the SIRI in patients with Hodgkin lymphoma. The optimal cutoff value was determined using the Youden index, and the area under the ROC curve (AUC) was reported along with the 95% confidence interval (CI).

Survival analyses for SIRI and the IPS-7 were carried out using the Kaplan–Meier method, and differences between survival curves were compared using the log-rank test. For survival analyses, SIRI was categorized using the statistically determined optimal cutoff value. Although SIRI is inherently a continuous biological variable, categorical classification was preferred to allow clinically interpretable survival comparisons. Prognostic factors affecting overall survival were evaluated using univariate and multivariate Cox proportional hazards regression analyses. The results of the Cox regression analyses were reported as hazard ratios (HR) with the corresponding 95% confidence intervals. All statistical tests were two-sided, and a *p*-value < 0.05 was considered statistically significant.

## 3. Results

The mean age of the 105 patients included in the study (57 men, 48 women) was 49 ± 18 years. 34 patients (32.4%) were in the early stage, and 71 patients (67.6%) were in the advanced stage.

The average follow-up period for patients was 45 ± 33 months (maximum 120 months).

According to the IPS-7 score, 62 patients (59%) were in the low-risk group, 34 patients (32.4%) were in the intermediate-risk group, and 9 patients (8.6%) were in the high-risk group. According to the IPS-3 score, 20 patients (19%) were in the low-risk group, 79 patients (75.2%) were in the intermediate-risk group, and 6 patients (5.7%) were in the high-risk group.

In risk stratification for early-stage disease, there were 10 patients (9.5%) in the early-stage favorable risk group, 24 patients (22.9%) in the early-stage unfavorable risk group, and 71 patients (67.6%) in the advanced-stage group. At least one comorbid condition was present in 40 patients (38.1%).

Overall, consolidative radiotherapy was administered to 10 patients (9.5%) in the study cohort.

In terms of treatment response, the majority of patients (*n* = 79, 75.2%) achieved complete response. However, during the follow-up period, disease relapse occurred in 26 patients (24.8%), and 29 patients (27.6%) died. These findings indicate that despite an initial treatment response, a substantial proportion of the study population experienced clinically meaningful events.

**Figure 1:**
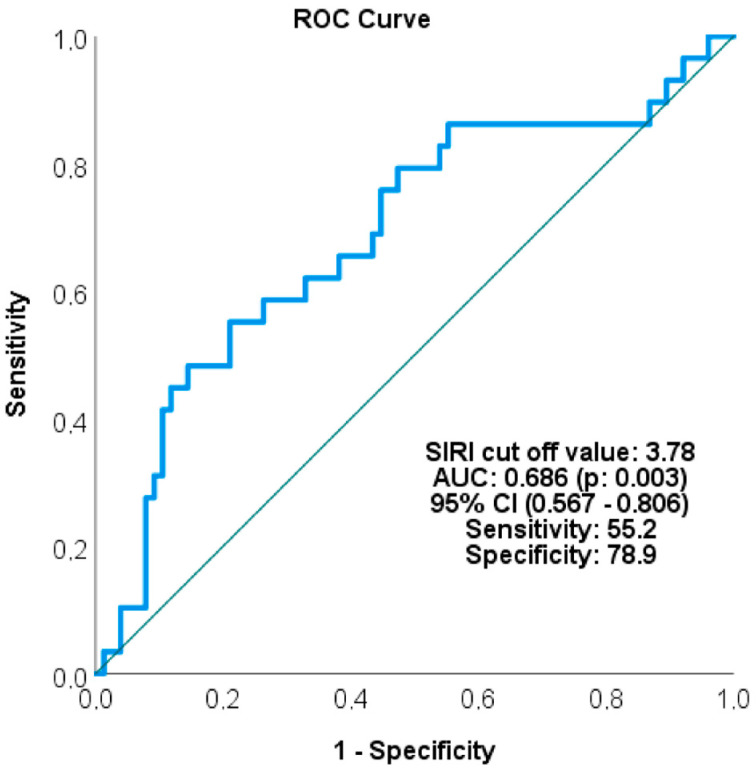
ROC analysis of the SIRI.

In the ROC analysis performed for survival prediction, the cutoff value for SIRI was determined to be 3.78. AUC = 0.686 (95% CI: 0.567–0.806, *p* = 0.003; sensitivity 55.2%, specificity 78.9%) (Figure 1). Patients were divided into two groups based on this value (<3.78 vs. ≥3.78).

SIRI was low (<3.78) in 73 patients (69.5%) and high (≥3.78) in 32 patients (30.5%).

### 3.1. Clinical Characteristics and Associations

We evaluated the relationship between SIRI levels and baseline clinical parameters to understand how systemic inflammation correlates with disease features. A SIRI ≥ 3.78 was associated with advanced stage, poor performance score, high IPS-7 and IPS-3 scores, no response or partial response to treatment, relapse, and increased mortality (Table 1).

When the relationship between SIRI and laboratory parameters was evaluated, patients with high SIRI levels exhibited higher white blood cell, neutrophil, monocyte, and platelet counts, along with lower lymphocyte counts, reduced hemoglobin and albumin levels, and elevated ESR (Table 2).

A positive correlation was found between SIRI and IPS-7 score in the correlation analysis (*p* < 0.001, rho = 0.355). This finding highlights that SIRI is associated with established prognostic scores.

### 3.2. Survival Analyses

We assessed the prognostic impact of SIRI and other clinical variables on PFS and OS. In the univariate analysis for PFS, Hb, IPS-7 score, and SIRI were identified as prognostic factors (*p* = 0.004, *p* = 0.000, *p* = 0.002, respectively). In the multivariate analysis, only high SIRI level was shown to be an independent prognostic factor for PFS (*p* = 0.033) (Table 3).

In the univariate analysis for OS, age, Hb, albumin, lymphocyte count, IPS-7 score, and SIRI were identified as prognostic factors (*p* = 0.025, *p* = 0.001, *p* = 0.001, *p* = 0.010, *p* = 0.000, *p* = 0.001, respectively). Multivariate analysis showed that age over 45 and high SIRI were independent prognostic factors for OS (*p* = 0.016, *p* = 0.012) (Table 4).

### 3.3. Kaplan–Meier Survival Analysis

**Figure 2:**
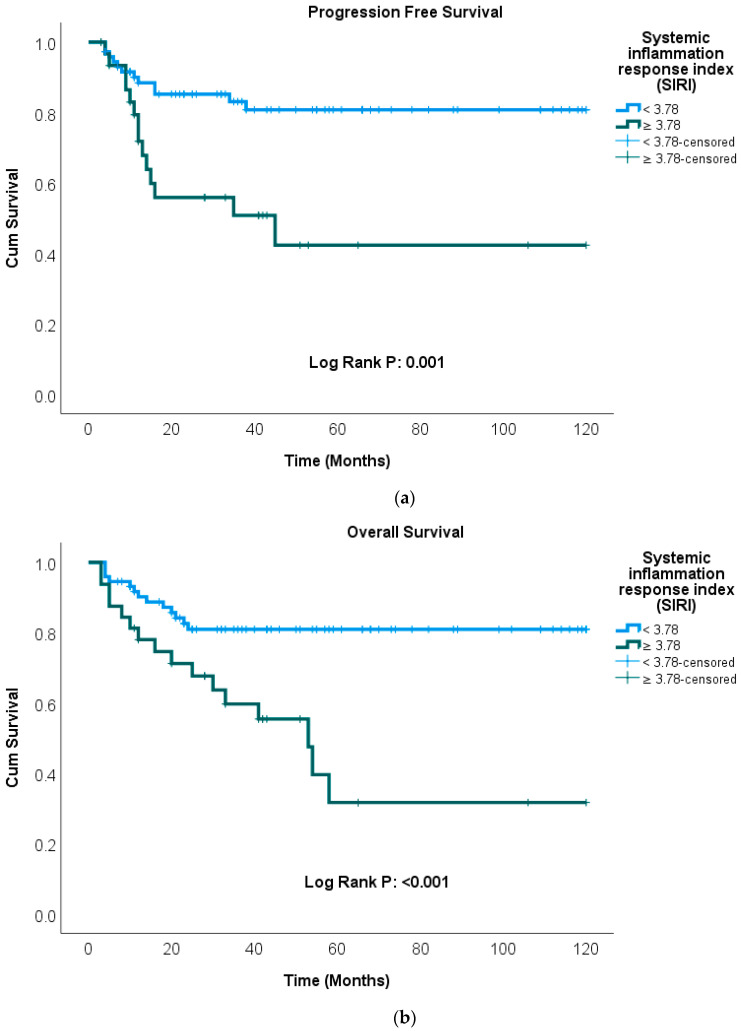
Kaplan–Meier analysis of progression-free survival (**a**) and overall survival (**b**) stratified by SIRI.

**Figure 3:**
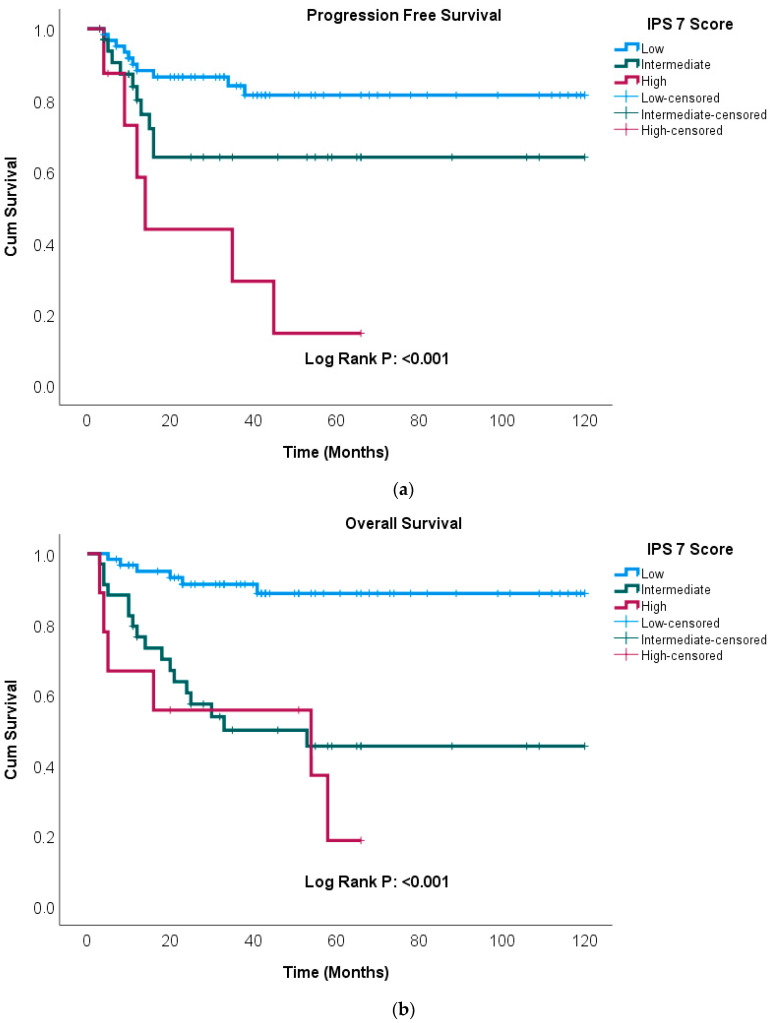
Kaplan–Meier analysis of progression-free survival (**a**) and overall survival (**b**) stratified by IPS-7 score.

Kaplan–Meier curves were generated to illustrate the effects of SIRI and IPS-7 on patient survival. Kaplan–Meier survival analysis demonstrated that patients with a high SIRI had significantly shorter PFS and OS (*p* = 0.001, *p* < 0.001) (Figure 2a,b). Additionally, patients with high IPS-7 scores exhibited significantly shorter PFS and OS (*p* < 0.001, *p* < 0.001) (Figure 3a,b).

The mean OS was 99.6 months (95% CI: 89.7–109.6) and the mean PFS was 99.8 months (95% CI: 89.5–110.2) in patients with SIRI < 3.78, whereas in those with SIRI ≥ 3.78, the mean OS and PFS were 58.9 months (95% CI: 39.8–78.0) and 61.2 months (95% CI: 39.7–82.8), respectively.

## 4. Discussion

This study has several strengths and limitations that should be considered when interpreting the results. The main strength is that it is the first study to evaluate the prognostic significance of the SIRI in patients with HL. In our cohort, high SIRI was identified as an independent prognostic factor and was associated with shorter progression-free survival and overall survival, and a positive correlation between SIRI and the IPS-7 score was also observed. However, the retrospective design of the study may limit causal inference. In addition, the single-center setting, the long study period, and the relatively limited sample size represent further limitations. Therefore, the results should be interpreted with caution, and multicenter prospective studies with larger patient populations are warranted to validate these findings.

It is thought that the poorer clinical outcomes in elderly HL patients compared to younger patients are due to multiple factors, including lower tolerance to modern intensive chemotherapy regimens, different disease biology, more advanced disease at diagnosis, and increased comorbidity burden [31,32]. Indeed, being over 45 years of age at diagnosis has been identified as an independent risk factor for poor prognosis within the IPS-7 [6]. In the study by Gao et al., cumulative mortality due to non-disease-related causes was shown to be significantly higher in HL patients over the age of 60 compared to younger patients [33]. In a study conducted by the German Hodgkin Study Group, the mortality rate in patients over 60 years of age treated with the ABVD regimen was found to be 28% at the end of a follow-up period of approximately 92 months, and the 5-year disease-free survival rate was reported to be 75% [34]. Similarly, another study emphasized that the mortality rate without progression increased significantly in older HL patients and that age-related survival differences in HL were largely due to non-lymphoma events [35]. Additionally, in a study including 269 HL patients aged 60 years or older, the 5-year PFS was reported as 52.2%, OS as 62.5%, and cause-specific survival as 85.1% during an average follow-up period of 6.6 years [36]. In this study, it was demonstrated that being over 45 years of age does not affect PFS, but is an independent prognostic factor in terms of OS. This finding indicates that, with advancing age in patients with HL, mortality attributable to non-disease-related causes plays an increasingly decisive role in clinical outcomes. It further underscores the importance of considering host related factors and comorbidities, in addition to tumor related parameters, when performing risk stratification, particularly in older patients.

The IPS-7 score is one of the most commonly used prognostic risk classification tools in clinical practice for advanced-stage HL patients, including seven clinical factors [7]. While IPS-7 is useful in identifying patients with poor prognosis, it has been reported that its prognostic discriminatory power has decreased with advances in treatment strategies and the addition of immunotherapies to standard treatments [3,4,9]. In a study by Moccia et al. including 740 HL patients, IPS-7 was shown to retain its prognostic value for both PFS and OS; however, the clinical discriminatory power between low- and high-risk groups was significantly reduced in patients treated with ABVD-like regimens [37]. Similarly, another study reported that the IPS-3 score, derived from IPS-7, provided better discriminatory power in predicting PFS and OS compared to IPS-7, despite containing fewer variables [7]. The limited ability of the IPS-7 to adequately discriminate risk groups in the modern treatment era, along with evidence demonstrating interim PET assessment as a more powerful prognostic factor, has increased the need for novel prognostic models reflecting inflammation and host immune status. Accordingly, research efforts aimed at developing inflammation-based indices have gained momentum [10,38,39]. Importantly, although SIRI was found to be associated with IPS-7 in our cohort, it retained its prognostic significance as an independent factor in multivariate analysis. Therefore, SIRI should be considered not as a surrogate or extension of IPS-7, but rather as an independent and complementary biomarker that captures biological aspects not fully addressed by traditional clinical risk scores.

Prognostic scores based solely on clinical and disease-specific variables may not adequately reflect the inflammatory and immunological nature of HL; therefore, biomarkers encompassing systemic inflammation and host immune status may play a complementary role to existing risk classification models. Our study demonstrated that high IPS-7 and IPS-3 scores were significantly associated with high SIRI levels. Patients with high IPS-7 scores had significantly shorter PFS and OS durations, and a positive correlation was found between SIRI and IPS-7 scores. The observed association between SIRI and IPS-7 in this study should not be interpreted as SIRI merely reflecting a retrospective prognostic model. While IPS-7 is primarily based on clinical and demographic variables, SIRI is a biological marker that reflects host-derived systemic inflammation and immune response. Therefore, its association with IPS-7 should be considered as an indicator of the interaction between tumor biology and host response, rather than a temporal dependency. These findings suggest that evaluating IPS-7 in conjunction with SIRI, which reflects the inflammatory burden, may provide a more accurate and comprehensive prognostic prediction compared to clinical scores alone.

Recent studies have revealed the decisive effects of local immune responses and systemic inflammatory processes on cancer progression, metastasis, and patient survival. Systemic inflammation includes T and B lymphocytes, monocytes, neutrophils, cytokines, and various inflammatory proteins that can be measured in the circulation, contributing to tumor development through the stimulation of angiogenesis, immune escape mechanisms, and genomic instability [17,40,41].

SIRI is a comprehensive parameter reflecting the systemic inflammatory response when neutrophil and monocyte counts are considered together with lymphocyte levels, and it is noteworthy for indirectly revealing the interaction between the tumor microenvironment and the immune response. Higher SIRI values indicate an increased neutrophil/monocyte burden and/or decreased lymphocyte count. In Hodgkin lymphoma, inflammation is pronounced due to the cytokine rich microenvironment generated by Reed Sternberg cells, and neutrophils as well as tumor-associated macrophages (TAM) derived particularly from monocytes play a critical role in shaping tumor behavior. Neutrophils increase angiogenesis by producing mediators such as transforming growth factor-beta (TGF-β), vascular endothelial growth factor (VEGF), matrix metalloproteinases (MMPs), and IL-6, IL-8, and IL-12. Additionally, MMP disrupts cell connections by producing proteases, including cysteine cathepsins and serine proteases, and breaks down extracellular matrix and basement membrane proteins, thereby facilitating tumor cell migration and supporting tumor cell invasion [42,43,44]. Similarly, the recruitment of monocytes to the tumor microenvironment and their conversion into TAMs is facilitated by the chemotactic effect mediated by tumor necrosis factor-α (TNF-α) and monocyte chemotactic protein-1 (MCP-1), and these cells constitute a dominant phenotype in progressive disease. Additionally, the inhibitory effects of monocytes on lymphocyte proliferation may weaken the antitumor immune response [45]. In contrast, lymphocytes are the fundamental components of an effective cell-mediated immune response. Low lymphocyte levels are associated with a more aggressive clinical course because they weaken the immune system and make it easier for tumor cells to escape immune surveillance [46]. In line with these mechanisms, the components of SIRI reflect the biological characteristics of the distinct inflammatory tumor microenvironment in Hodgkin lymphoma, and the increase in index levels suggests an imbalance in the inflammation-immune response. Additionally, although inflammation-based indices such as NLR, LMR, and systemic immune-inflammation index (SII) have been previously studied in HL, the SIRI offers certain conceptual advantages [14,16,21]. SIRI provides a more comprehensive representation of multi-cellular interactions and tumor-microenvironment relationships and, compared to indices based on only two cell types such as NLR or LMR, it has the potential to more accurately and holistically classify patients’ systemic inflammatory status. The inclusion of monocytes allows SIRI to capture contributions from monocyte-derived cells involved in tumor-promoting and immunoregulatory processes, thereby reflecting a broader aspect of systemic inflammation and potentially enhancing its prognostic relevance relative to SII and other two-cell indices.

In a study conducted by Jiang et al. in patients with lung adenocarcinoma, high SIRI and poor performance score were shown to independently predict PFS and OS [25]. A study conducted on breast cancer patients receiving neoadjuvant chemotherapy found that low SIRI was associated with a better prognosis and lower recurrence rates [26]. Another study found that preoperative inflammatory indices and SIRI were independent prognostic factors in patients with non-metastatic renal cell carcinoma and were also associated with advanced stage and large tumor size [47].

In a study of patients diagnosed with diffuse large B-cell lymphoma of the gastrointestinal tract, a pre-treatment SIRI ≥ 1.34 was identified as an independent prognostic factor associated with poorer survival [22]. Similarly, in patients with peripheral T-cell lymphoma, high SIRI has been shown to be an independent risk factor affecting treatment efficacy, and in patients with primary central nervous system lymphoma, being under 65 years of age and having low SIRI prior to treatment have been shown to independently predict longer OS [23,24]. The demonstration that SIRI is a prognostic marker in many malignancies, regardless of whether they are solid tumors or non-Hodgkin lymphoma, has once again shown that disease-related inflammation plays an important role in treatment response and disease course.

In this study, high SIRI was identified as an independent prognostic factor and was associate with short PFS and OS. This result indicates that the balance between systemic inflammation and host immune response plays an important role in determining disease course in HL. From a clinical perspective, the course of the disease appears to be closely related not only to static variables such as stage and tumor burden, but also to dynamic host response indicators that can be measured at the time of diagnosis. Being an easily measurable and reproducible marker makes SIRI suitable for use in daily clinical practice. It should be considered as a complementary biomarker for risk stratification in clinical settings where advanced molecular analyses are not readily available. Its evaluation in conjunction with existing prognostic models may contribute to the early identification of biologically more aggressive patient subgroups that cannot be adequately explained by classic clinical variables. In this context, SIRI has the potential to serve as a guiding tool for clinicians in developing risk-adapted treatment strategies.

### Future Directions

Future prospective studies including large patient cohorts may more clearly demonstrate the predictive value of SIRI in subgroups of patients with poor prognosis in HL. Especially in relapsed/refractory disease and in patient groups treated with new-generation therapeutic agents such as immune checkpoint inhibitors and brentuximab vedotin, dynamic assessment of SIRI before treatment, at the onset of relapse, and after treatment may provide meaningful contributions in predicting treatment response. Examining the relationship between in SIRI and treatment efficacy, resistance development, and survival outcomes may enable the assessment of its prognostic potential in relapsed refractory disease and may be considered as an factor in the design of future clinical trials.

## 5. Conclusions

A high SIRI prior to treatment was identified as an independent prognostic factor in HL patients and was associated with shorter PFS and OS. Despite its positive correlation with the IPS-7 score, SIRI retained its independent prognostic value and should be considered a biomarker that provides complementary biological information to existing clinical risk scores. This cost-effective, noninvasive, and easily accessible index can contribute to the prediction of high-risk patients and assist clinicians in their decision-making processes by enabling individualized risk assessment.

## Figures and Tables

**Table 1:** Comparison of sociodemographic and clinical characteristics between SIRI groups.

		SIRI		
		<3.78	≥3.78	
		*n* (%)	*n* (%)	*p*
Age (years)	<45	27 (37)	15 (46.9)	0.341
	≥45	46 (63)	17 (53.1)	
Gender	Female	32 (43.8)	16 (50)	0.559
	Male	41 (56.2)	16 (50)	
Stage	1 + 2	28 (38.4)	6 (18.8)	0.048
	3 + 4	45 (61.6)	26 (81.3)	
ECOG PS	0 + 1	72 (98.6)	24 (75)	<0.001
	2 + 3	1 (1.4)	8 (25)	
Bulky Disease	None	70 (95.9)	29 (90.6)	0.366
	Yes	3 (4.1)	3 (9.4)	
IPS 7 Score	Low	49 (67.1)	13 (40.6)	0.002
	Intermediate	22 (30.1)	12 (37.5)	
	High	2 (2.7)	7 (21.9)	
IPS 3 Score	0	15 (20.5)	5 (15.6)	0.047
	1	37 (50.7)	9 (28.1)	
	2	17 (23.3)	16 (50)	
	3	4 (5.5)	2 (6.3)	
Risk group	Early stage favorable risk	9 (12.4)	1 (3.1)	0.117
	Early stage unfavorable risk	19 (26)	5 (15.6)	
	Advanced stage	45 (61.6)	26 (81.3)	
Comorbidity	None	46 (63)	19 (59.4)	0.724
	Yes	27 (37)	13 (40.6)	
Treatment response	No/Partial response	12 (16.4)	14 (43.8)	0.003
	Complete response	61 (83.6)	18 (56.3)	
Relapse status	None	61 (83.6)	18 (56.3)	0.003
	Yes	12 (16.4)	14 (43.8)	
Survival status	Survivor	60 (82.2)	16 (50)	<0.001
	Non-survivor	13 (17.8)	16 (50)	

ECOG PS: Eastern Cooperative Oncology Group performance status; IPS: International Prognostic Score.

**Table 2:** Baseline laboratory parameters by SIRI groups.

	SIRI		
	<3.78	≥3.78	
	Mean ± SD Median (Min–Max)	Mean ± SD Median (Min–Max)	*p*
Wbc (10^3^/μL)	6.82 (2.5–26.18)	11.6 (4.1–28.8)	<0.001
Neutrophil (10^3^/μL)	4.43 (1.2–19.75)	9.1 (3.02–25.83)	<0.001
Monocyte (10^3^/μL)	0.57 (0.03–2.47)	0.96 (0.21–4.03)	<0.001
Lymphocyte (10^3^/(μL)	1.78 (0.35–12.17)	1.51 (0.27–5.65)	0.035
Hemoglobin (gr/dL)	12.9 ± 1.8	11.5 ± 2	<0.001
Platelet (10^3^/μL)	297 ± 105	367± 161	0.029
Albumin (g/dL)	4.2 (3.2–5.1)	3.8 (2.4–4.5)	0.001
D. bilirubine (mg/dL)	0.1 (0–0.8)	0.1 (0.1–0.7)	0.096
ESR (mm/h)	19 (2–111)	50 (9–110)	<0.001

Wbc: White blood cell; D.: Direct; ESR: Erythrocyte sedimentation rate.

**Table 3:** Independent prognostic factors associated with progression free survival in cox regression model.

	Univariate Analysis				Multivariate Analysis			
	*p*	HR	95% CI for HR		*p*	HR	95% CI for HR	
			Lower	Upper			Lower	Upper
Gender	0.168	1.796	0.781	4.131				
Age (years)	0.939	0.970	0.445	2.114				
Stage	0.082	2.020	0.916	4.454	0.440	1.415	0.587	3.410
Hemoglobin(gr/dL)	0.004	3.498	1.507	8.117	0.159	1.986	0.764	5.162
Albumin (g/dL)	0.055	0.469	0.217	1.015	0.688	0.835	0.347	2.011
Wbc (10^3^/μL)	0.991	0.992	0.234	4.201				
Lymphocyte (10^3^/μL)	0.411	1.834	0.432	7.789				
IPS 7 Score (intermediate vs. low)	0.069	2.258	0.938	5.432				
IPS 7 Score (high vs. low)	0.000	6.521	2.361	18.011				
SIRI	0.002	3.359	1.548	7.293	0.033	2.516	1.077	5.881

Wbc: White blood cell; IPS: International Prognostic Score; SIRI: Systemic inflammation response index.

**Table 4:** Independent prognostic factors associated with overall survival in cox regression model.

	Univariate Analysis				Multivariate Analysis			
	*p*	HR	95% CI for HR		*p*	HR	95% CI for HR	
			Lower	Upper			Lower	Upper
Gender	0.655	1.184	0.565	2.480				
Age (years)	0.025	2.800	1.139	6.882	0.016	3.155	1.241	8.018
Stage	0.086	1.929	0.911	4.087	0.770	1.135	0.485	2.657
Hemoglobin(gr/dL)	0.001	3.541	1.642	7.637	0.127	2.038	0.816	5.090
Albumin (g/dL)	0.001	0.288	0.133	0.619	0.175	0.537	0.218	1.318
Wbc (10^3^/μL)	0.631	1.340	0.405	4.430				
Lymphocyte (10^3^/μL)	0.010	4.084	1.408	11.840	0.414	1.674	0.486	5.773
IPS 7 Score (intermediate vs. low)	0.000	6.237	2.456	15.838				
IPS 7 Score (high vs. low)	0.000	9.356	3.013	29.048				
SIRI	0.001	3.316	1.588	6.923	0.012	2.731	1.251	5.964

Wbc: White blood cell; IPS: International Prognostic Score; SIRI: Systemic inflammation response index.

## Data Availability

The data are available from the corresponding author upon reasonable request.
